# Effects of Suppression and Expression of Academic Emotions on Peer Acceptance in Outperformance and Underperformance Situations

**DOI:** 10.3390/bs15101366

**Published:** 2025-10-07

**Authors:** Ying Liu, Biao Sang

**Affiliations:** 1 Wenbo College, East China University of Political Science and Law, Shanghai 200042, China; 2 Lab for Educational Big Data and Policymaking, Ministry of Education, Shanghai Academy of Educational Sciences, Shanghai 200032, China

**Keywords:** adolescents, academic emotion, emotional suppression, emotional expression, outperformance, underperformance

## Abstract

The current study was conducted to investigate the cross-situational differences in the effect of the suppression and expression of academic emotions on peer acceptance in situations involving outperformance and underperformance. A total of 81 adolescents were randomly selected to evaluate a target classmate’s acceptance level when underperforming or outperforming in a predetermined hypothetical setting using two questionnaires. The results obtained from the paired sample *t*-test showed that the relationship between the suppression or expression of academic emotions and peer acceptance has situational specificity; that is, compared with adolescents expressing positive academic emotions when outperforming others, adolescents expressing negative academic emotions when underperforming achieve higher levels of peer acceptance. In addition, in outperformance, peer acceptance was higher when positive academic emotions were suppressed rather than expressed; in underperformance, acceptance was significantly higher when negative academic emotions were suppressed rather than expressed. These findings underscore the significance of situations involving outperformance and underperformance in shaping the effectiveness of academic emotion regulation strategies, and support the different adaptive values of emotional expression and expressive suppression in both types of situations.

## 1. Introduction

Learning is an activity accompanied by numerous emotions (Schutz et al., 2006). Students experience a variety of them (e.g., happy, anxiety) after learning of academic success or failure, during classroom learning activities and completion of tasks, and during and after examinations (Pekrun et al., 2002; Yu & Dong, 2005). These emotional experiences have a significant impact on the individual’s learning behavior and outcomes (Forsblom et al., 2022; Lichtenfeld et al., 2023; Wang et al., 2022; Zhen et al., 2017). Regulating and managing emotional experiences effectively in academic settings is, therefore, necessary in order to orient emotional experiences to benefit students’ learning. In the area of emotion regulation, various strategies are available, among them emotional expression and expressive suppression, both of which are response-focused strategies (J. J. Gross, 1989, 1998). Specifically, the expression of academic emotions refers to the conscious revelation of one’s academic emotions while academic emotionally aroused; and the suppression of academic emotional expression refers to the conscious inhibition of one’s own academic emotionally expressive behavior while academic emotionally aroused.

Previous studies have shown that emotional expression and expressive suppression are considered to be a more interpersonal form of emotion regulation than other forms of regulation strategies because they point directly to external expressive behaviors that are visible to others (Impett et al., 2012). Relevant empirical research results also have shown that the use of strategies for emotional expression and expressive suppression can affect individuals in social ways (Cameron & Overall, 2018; Liu et al., 2019); however, researchers have primarily investigated this topic from a holistic standpoint with less emphasis on the importance of specific situations when choosing strategies for suppressing or expressing emotions, especially academic emotions. In contrast to whether people express or suppress their emotions, an increasing number of emotional theory researchers have concluded that the capacity to express or suppress emotions flexibly in response to situational needs is more crucial for obtaining adaptive outcomes (Double et al., 2022; J. T. Gross & Cassidy, 2019). Thus, investigating emotional expression and expressive suppression as variable strategies is necessary to elucidate the role of situation in their use. To better understand the effects of emotional expression and expressive suppression on Chinese adolescents in situations where they outperformed and underperformed with respect to others, we focused primarily on the relationship between these two strategies and peer acceptance.

### 1.1. The Role of Situation in the Effect of Expression and Expressive Suppression of Academic Emotions

Numerous researchers have revealed that expressive suppression is not always maladaptive (Liu et al., 2016; Paul et al., 2023) and that emotional expression is not always adaptive (Lange & Crusius, 2015). People regulate their emotions not only for hedonic reasons (e.g., up-regulating positive emotions to make themselves feel better) but also for practical purposes (e.g., because certain emotions may help them achieve their goals); therefore, they may engage in the regulation of contrahedonic emotions based on the situation (e.g., down-regulating positive emotions), focusing on matching emotional experience with the situation (Greenaway & Kalokerinos, 2017). The broad description of the expression of emotions as good and the expressive suppression as bad is largely the result of a failure to recognize the role of situation. For example, some previous researchers have linked the expression of positive emotions to personal and social benefits (e.g., reduce negative academic emotions, improve well-being, and maintain relationships (Greenaway & Kalokerinos, 2017; Harker & Keltner, 2001; Liu, 2018); however, others have found that in teaching situations, when teachers expressed happiness, students’ academic performance was poorer than when teachers expressed anger (Van Doorn et al., 2014). Compared with expressing negative academic emotions, suppressing negative academic emotions in unpleasant academic situations can lead to better academic performance among adolescents (Liu et al., 2018). Suppressing positive emotions in competitive situations, results in higher levels of peer acceptance, while in cooperative situation expressing the positive emotions increase peer acceptance (Liu et al., 2024a).

The results noted above validated the core emphasized in strategy–situation fit theory from the perspective of flexibility in emotion regulation, namely that no single strategy for the regulation of emotion is generally beneficial or harmful and that the effects of any such strategy depend on the situation. When individuals can select and apply the appropriate strategy based on the demands of the situation, the strategy can have adaptive benefits; otherwise, it will have a negative impact (Aldao, 2013; Aldao et al., 2015; Bonanno & Burton, 2013; Haines et al., 2016). Results of previous researchers also adhered to the principle of emotional mismatch, proponents of which have argued that when emotional expression matches the situation, social interaction is likely to proceed smoothly. When the emotions expressed are incongruous with the situation, a negative impact on individuals can occur, particularly in terms of social interaction (Greenaway & Kalokerinos, 2017). At the same time, these results also emphasize the necessity of examining the situational specificity of the effect of academic emotional expression and expressive suppression.

#### 1.1.1. Differences in the Effect of Expression and Expressive Suppression of Academic Emotions When Outperforming Others and Underperforming

Adolescents may experience not only a range of situations involving emotional arousal but also those involving the regulation of academic emotion in the learning process. In the face of situations involving the latter, they may adopt various strategies for expressing emotion to control their responses and to cope with a challenging environment or the people in it (Kurki et al., 2014), ultimately serving their learning and social interaction.

Competition is a goal-oriented behavior aimed at achieving relative success or comparative advantage (Deutsch, 2005); school as a social subsystem likewise involves competition. The full spectrum of the competitive scenarios in which adolescents find themselves ranges from the considerable competition among students taking high school and college admission exams to the less intense competition among students merely completing assignments. Parties are opposed when in competition. When one side has a higher chance of success, the other party has a lower chance of success (Deutsch, 2005). In other words, competition is “exclusive” because it depends on one party defeating the other for a winner to emerge (De Dreu, 2010). Notably, comparison occurs whenever competition exists. In competitive activities people frequently assess their performance in comparison to their rivals. Then, based on performance in the competitive activities, people find themselves in positions of outperformance and underperformance. Individuals are in an outperformance position when they exceed their opponents in accomplishing their goals. When individuals perform worse in achieving their goals than their competitors, they are in a position of underperformance (Schall et al., 2016).

Researchers have studied how adolescents’ social outcomes are affected by academic emotional expression and expressive suppression, finding that in competitive situations adolescents who suppressed their positive or negative academic emotions also scored higher in terms of peer acceptance than those who expressed their feelings (Liu et al., 2024a). Moreover, previous researchers have demonstrated that emotional expression and expressive suppression have varying effects on peer acceptance in situations of both outperformance and underperformance; for example, research on adults showed that people thought winners who expressed positive emotions were more arrogant and were viewed less favorably than winners who suppressed positive emotions (Kalokerinos et al., 2014). Similarly, research on students in Grades 10 and 11 showed significantly lower peer acceptance of target students who expressed positive emotions in outperformance compared to nonoutperformance (Schall et al., 2016). Although researchers have explored situational differences (outperformance and underperformance) in the relationship between the expression and expressive suppression of academic emotions and social outcomes, relevant studies have focused either on situations of outperformance in daily life or on the expression and expressive suppression of positive emotions in situations of outperformance. In the division of competitive situations like outperformance and underperformance, one still finds few studies on the impact of adolescents’ academic emotional expression and expressive suppression on peer acceptance. Significant theoretical relevance and practical benefit will result from further investigation of this matter.

#### 1.1.2. Differences in the Relationship Between Expression and Expressive Suppression of Academic Emotions and Peer Acceptance in Outperformance and Underperformance in China

Peer acceptance is the degree to which a person is liked or accepted by other members of the peer group (Parker & Asher, 1993; Zou, 1997). Likability denotes the extent to which an individual is perceived as favorable or agreeable by others, whereas acceptance refers to the extent to which an individual is recognized and included by others (Schall et al., 2016). The reason for focusing on peer acceptance is that from a socioemotional perspective, adolescents become increasingly dependent on peer groups while their need for parental support eventually declines (Roach, 2018). Establishing good peer relationships has also become one of the most important developmental tasks for adolescents in a period of dramatic emotional and social transformation (Brown & Larson, 2009); moreover, peer acceptance as an important manifestation of social outcomes among adolescents is closely related to their physical and mental health (Wu et al., 2019) as well as academic achievements (Kyongboon et al., 2022; Li & Wang, 2022; Oberle & Schonert-Reichl, 2013).

The focus on Chinese adolescents stems from the fact that especially in Chinese culture the idea that “knowledge changes destiny” is deeply rooted in the hearts of the people, and they have become increasingly concerned about college admission exams as a “stepping stone” to higher education, a desired goal (Zheng & Chen, 2013). Parents regard their children as the future and hope of the family, even the “face” of the family, and expect their children to excel through education (Niu et al., 2023); however, China’s college admission exams, including the high school admission exams, are highly competitive. Under the baton effect of the high school and college admission exams, Chinese adolescents, therefore, must endure the pressure of academic competition (Kipnis, 2011). In this context of fierce academic competition, adolescents frequently experience outperformance or underperformance. How can they employ appropriate academic emotion regulation strategies to obtain better peer relationships? Current research in this area rare not fully understood. The relationship between expression and expressive suppression of academic emotions and peer acceptance must, therefore, be examined from the viewpoints of Chinese adolescents in situations of outperformance and underperformance because this may be more focused and useful.

### 1.2. Current Study

In the current study, we aimed to explore cross-situational differences in the effect of adolescents’ expression and expressive suppression of academic emotions on peer acceptance in outperformance and underperformance. Based on previous research, specific hypotheses for the current study were proposed:

**H1.**  *When outperforming, adolescents who use expressive suppression achieve a higher level of peer acceptance.*

**H2.**  *When outperforming those who use emotional expression reach a lower level of peer acceptance.*

**H3.**  *When underperforming, those who use emotional expression reach a higher level of peer acceptance.*

**H4.**  *When underperforming, those who use expressive suppression reach a lower level of peer acceptance.*

## 2. Method

### 2.1. Participants

Eighty-one adolescents in Grades 7 through 12 (age range = 12–17 years; *M*_age_ = 15.01 years, *SD* = 1.44 years; 42 boys, 39 girls) from two randomly selected, public, regular schools using electronic advertisements were chosen as participants. Over 90% of them were of Han nationality, the majority ethnic group in China, and 39.5% of them were from single-child families. 45.7% of their mothers and 44.4% of their fathers have completed high school or above. Sensitivity analyses with G*Power 3.1 showed our sample size to be sufficiently powered at 95% for detecting medium effects (Cohen’s d = 0.41) (Faul et al., 2009).

### 2.2. Measures

Regarding the evaluation of the target classmate’s acceptance level, participants were individually instructed to evaluate it on their own in both outperformance and underperformance in a predetermined hypothetical setting.

The experiment was split into two sections: First, the experimenter described the situation that the participants would face, which included the target classmate who (a) formed a relationship with them in which the target was outperforming or underperforming and (b) suppressed or expressed positive or negative academic emotions in their presence. For example, “Imagine now that you encounter a situation: On a recent exam, the target classmate did better than you, scored higher than you, and was praised and rewarded by the teacher, but you were not. The target classmate suppressed/expressed positive academic emotions (e.g., happiness, pride) in your presence, for example, not showing/showing you the happiness felt at the moment.” “Imagine now that you encounter a situation: On a recent exam, the target classmate did worse than you, scored lower than you, and you were praised and rewarded by the teacher, but the target classmate was not. The target classmate suppressed/expressed negative academic emotions (e.g., anxiety, frustration) in your presence, for example, not showing/showing you the anxiety felt at the moment.” The participants were then asked to rate their acceptance of the target classmate in the order of the situations described above. The level of a person’s likeability or acceptance among their peers is referred to as “peer acceptance” (Parker & Asher, 1993; Zou, 1997), gauged in the current study using the two variables: likability and acceptance.

#### 2.2.1. Likability

A likability questionnaire (Schall et al., 2016) which originally developed by Chaiken and Eagly (Chaiken & Eagly, 1983) was used to evaluate the likability of the target classmate after using strategies for suppressing or expressing academic emotions. The questionnaire consisted of five items (e.g., “I think this classmate is friendly”). Response options ranged from 1 (completely disagree) to 7 (completely agree). A total score was computed by averaging the items with higher values indicating higher likability. In outperformance and underperformance in the current study, Cronbach’s α for this questionnaire fell between 0.93 and 0.96 under the various conditions of strategies for suppressing and expressing academic emotions. This questionnaire has been previously used and shown to be valid for Chinese adolescents (Liu et al., 2024a, 2024b).

#### 2.2.2. Acceptance

An acceptance questionnaire (Schall et al., 2016) which originally developed by Wentzel (Wentzel, 1994) (three items, e.g., “I would like to engage with this classmate in school activities,” “I would like this classmate to be in my class,” and “I would like to have this classmate as coworker”, rated on a 7-point scale) was used to evaluate the acceptance of the target classmate after using strategies for suppressing or expressing academic emotions. A higher average score indicated a higher degree of acceptance by others. In the outperformance and underperformance in the current study, Cronbach’s α for this questionnaire fell between 0.90 and 0.93 under the various conditions of strategies for suppressing and expressing academic emotions. The questionnaire has been demonstrated to be a reliable measurement for Chinese adolescents (Liu et al., 2024a).

### 2.3. Procedure

Graduate psychology students who had undergone training administered the master test. Individual testing is conducted after securing permission from parents and the adolescents themselves, and the questionnaire was collected immediately after completion, which took around 8 min. The collected data are stored in encrypted digital files on the computer, accessible only to the principal investigator. A physical copy of the raw data will be securely stored in a lockable cabinet within the laboratory, the key to which is retained by the principal investigator. Upon completion of data analysis, participants will receive a summary report of the research findings, and specialized lectures will be conducted for students to support the application of theoretical knowledge in practical contexts.

### 2.4. Analytic Strategy

Paired samples *t*-tests were conducted on the data using SPSS 20.0 software.

## 3. Results

### 3.1. Preliminary Analyses

The means, standard deviations, and correlation matrix of variables are presented in Table 1. Likability after suppression of positive academic emotions was positively correlated with acceptance after suppression of those emotions. Similarly, likability after expression of positive academic emotions showed a positive correlation with acceptance after expressing both positive and negative academic emotions. Furthermore, likability after suppression of negative academic emotions was positively associated with acceptance after suppression of those emotions. In addition, likability after expression of negative academic emotions was positively correlated with acceptance after their expression. Acceptance after suppression of positive academic emotions was also positively correlated with acceptance after expression of those emotions. Lastly, acceptance after expression of positive academic emotions was positively correlated with acceptance after expression of negative academic emotions.

### 3.2. Relationship Between Expression and Expressive Suppression of Academic Emotions and Likability in Outperformance and Underperformance

First, we used a paired samples *t*-test to analyze the likability of the target individual after expressive suppression when positive and negative academic emotions were experienced in outperformance and underperformance, respectively. The results showed no significant difference in likability after expressive suppression in the two situations (*M* = 4.77, *SD* = 1.41; *M* = 4.80, *SD* = 1.29), *t* (80) = −0.22, *p* > 0.05, Cohen’s d = 0.02, 95%CI = [−0.41, 0.33].

Second, we analyzed the likability of the target individual after expressing positive and negative academic emotions in the outperformance and underperformance, respectively. The results of paired samples *t*-test showed a significant difference in likability after the expression of emotion in the two situations, *t* (80) = −3.48, *p* < 0.01, Cohen’s d = 0.48, 95%CI = [−1.16, −0.32]. Compared to the expression of positive academic emotions (*M* = 3.92, *SD* = 1.62), the target individual may achieve a higher level of likability when expressing negative academic emotions (*M* = 4.66, *SD* = 1.46).

**Figure 1:**
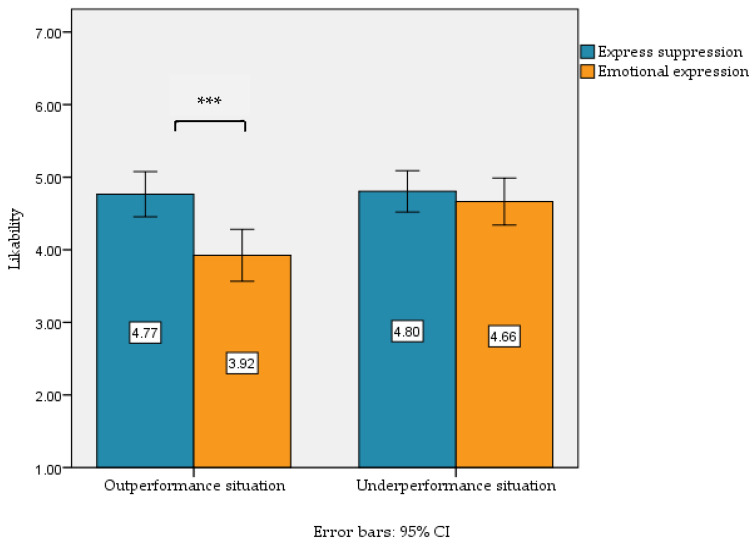
Likability after expression or expressive suppression of positive or negative academic emotions in outperformance and underperformance. Note. *** *p* < 0.001.

Finally, a paired samples *t*-test was conducted to examine the likability of the target individual after suppressing and expressing positive and negative academic emotions in both outperformance and underperformance. The results showed that when experiencing positive academic emotions in outperformance, the target individual may have lower levels of likeability after expressing positive academic emotions (*M* = 3.92, *SD* = 1.62) than after suppressing positive emotional (*M* = 4.77, *SD* = 1.41), *t* (80) = 3.69, *p* < 0.001, Cohen’s d = 0.56, 95%CI = [0.39, 1.30]. When negative academic emotions were experienced in underperformance, the difference in likability after expressive suppression (*M* = 4.80, *SD* = 1.29) and emotion expression (*M* = 4.66, *SD* = 1.46) was not significant, *t* (80) = 0.66, *p* > 0.05, Cohen’s d = 0.10, 95%CI = [−0.29, 0.57]. See Figure 1.

### 3.3. Relationship Between Expression and Expressive Suppression of Academic Emotions and Acceptance in Outperformance and Underperformance

First, we conducted a paired samples *t*-test to investigate acceptance of the target individual after expressive suppression when positive and negative academic emotions were experienced in outperformance and underperformance, respectively. The results showed no significant difference in acceptance after expressive suppression in the two situations (*M* = 4.78, *SD* = 1.66; *M* = 5.00, *SD* = 1.30), *t* (80) = −1.01, *p* > 0.05, Cohen’s d = 0.15, 95%CI = [−0.64, 0.21].

Second, we analyzed the acceptance of the target individual after expressing positive and negative academic emotions in outperformance and underperformance, respectively. The results of the paired samples *t*-test showed a significant difference in acceptance after the expression of emotion in the two situations, *t* (80) = −2.08, *p* < 0.05, Cohen’s d = 0.27, 95%CI = [−0.83, −0.02]. The acceptance of the target individual who expressed negative academic emotions (*M* = 4.53, *SD* = 1.49) was significantly higher than that of the one who expressed positive academic emotions (*M* = 4.10, *SD* = 1.74).

**Figure 2:**
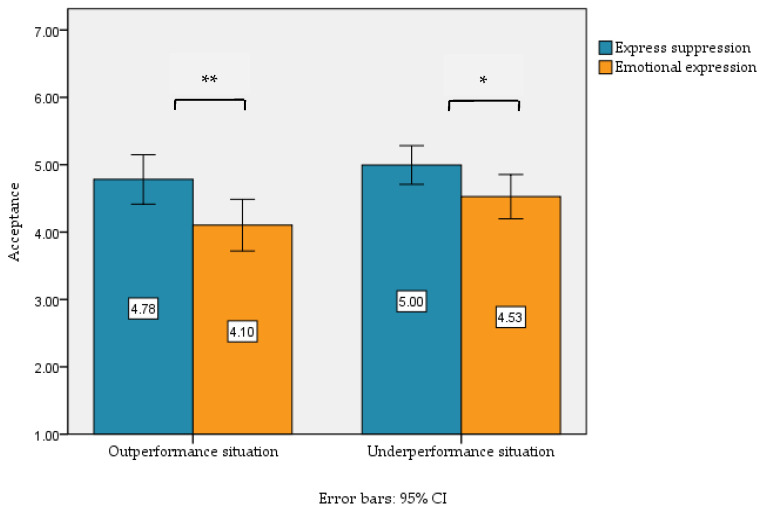
Acceptance after expression or expressive suppression of positive or negative academic emotions in outperformance and underperformance. Note. ** *p* < 0.01; * *p* < 0.05.

Finally, a paired samples *t*-test was conducted to examine the acceptance of the target individual after suppressing and expressing positive and negative academic emotions in both outperformance and underperformance. The results showed that when experiencing positive academic emotions in outperformance, acceptability after expressing positive academic emotion (*M* = 4.10, *SD* = 1.74) was significantly lower than that after suppressing it (*M* = 4.78, *SD* = 1.66), *t* (80) = 2.90, *p* < 0.01, Cohen’s d = 0.40, 95%CI = [0.21, 1.14]. When negative academic emotions were experienced in underperformance, acceptance after expressive suppression (*M* = 5.00, *SD* = 1.30) was significantly higher than that after expression (*M* = 4.53, *SD* = 1.49), *t* (80) = 2.40, *p* < 0.05, Cohen’s d = 0.34, 95%CI = [0.08, 0.86]. See Figure 2.

## 4. Discussion

### 4.1. Relationship Between Adolescents’ Expression and Expressive Suppression of Academic Emotions and Peer Acceptance in Outperformance and Underperformance

Through the integration of the results of the relationship between expression and expressive suppression of academic emotions and likability as well as the relationship between these strategies and acceptance, we found no significant difference in the level of peer acceptance in the current study after suppressing positive and negative academic emotions in outperformance and underperformance, respectively. The levels of peer acceptance following the expression of negative academic emotions, however, were significantly higher than those following the expression of positive academic emotions. According to social comparison theory, all people, as social beings, frequently compare themselves to others spontaneously and initiatively assess their own abilities based on the comparison (Festinger, 1954; Xie & Lu, 2014). When the evaluated party has outperformed and the assessor has underperformed, the former may signal that others are underperforming and show haughtiness, arrogance, and vanity if they express their positive academic emotions—particularly hubristic pride—to people who perform less well (Tracy & Robins, 2007, 2014). Previous researchers have also shown that when people compare themselves to and compete with others from similar backgrounds, those who underperform are more likely to feel envious of and dislike (e.g., hostility, resentment) those who outperform them (Feather & Sherman, 2002; Smith et al., 1990; Yang & Zhang, 2008), which can lead to behaviors like rejecting the outperforming others (Garcia et al., 2010). In contrast, when the evaluator outperforms and the assessed individual underperforms, the latter may show anxiety, frustration, and inferiority to the other person if expressing negative academic emotions to individuals who perform better than they do (Lockwood & Kunda, 1999; Zhang, 2012). At this point the evaluator who outperformed may be complacent and feel grateful and satisfied (Chen, 2012) as well as show comfort, support, and understanding to underperforming others (Sarason et al., 1990).

In addition, consistent with the findings of previous researchers (Kalokerinos et al., 2014; Schall et al., 2016), we found that in outperformance, the level of peer acceptance after suppressing positive academic emotions was higher than that after expressing positive academic emotions. Generally speaking, people who achieve success in a competitive situation will feel a sense of contradiction (Horner, 1968), and the winner is well aware that the results brought by outperformance are mixed (Exline & Zell, 2012). If the positive emotions brought about by successful competition are not expressed properly, they may lead to negative interpersonal consequences (such as being rejected) (Exline & Zell, 2012; Kalokerinos et al., 2014) because expressing emotions that match the emotions of interacting partners is crucial in the formation and maintenance of relationships (Anderson et al., 2003). In the context of social comparison, however, people often experience emotional mismatches with their comparison partner. For example, in the case of downward comparison, winners may perceive positive emotions, and their peers may perceive negative emotions (Tesser, 1988). On one hand, if the winners suppress the expression of positive emotions (i.e., pride), they can form an external expression of emotional consistency with their interacting partners, protect their feelings, and create a good peer relationship (Friedman & Miller, 1991; Kalokerinos et al., 2014; Mendolia et al., 1996). On the other hand, expressing one’s positive emotions to interacting partners may be evaluated as inappropriate by the other party (Kalokerinos et al., 2017). For example, individuals who express hubristic pride may be seen as attempting to secure their status through domination and control over others (Cheng et al., 2010).This type of behavior prioritizes one’s own needs while disregarding the feelings of others, ultimately damaging social relationships and undermining their likability (Cheng et al., 2013; Kalokerinos et al., 2014; Schall et al., 2016).

Finally, in the current study we found that in underperformance, acceptance after suppressing negative academic emotions was significantly higher than after expressing them. Researchers have found that emotions can be contagious (Hatfield et al., 1993). If individuals suppress the expression of negative emotions, they may reduce the negative emotions experienced by others (Nezlek & Kuppens, 2008). This view also applies to academic situations; that is, if adolescents who underperform act as an emotional sender and share their negative academic emotions with others who perform better—even if they receive comfort and understanding from the emotion receiver—doing so may also cause the receiver to experience negative academic emotions and other negative feelings. The threat of these negative emotional experiences to the ultimate learning goal is that the receiver will not make positive comments about the sender but will generate antipathy and rejection (Van Kleef, 2010). Notably, this difference has not been reflected in likability. Given the lack of relevant research, more empirical evidence is needed to test the reliability of this conclusion in the future.

### 4.2. Implications and Future Directions

Based on strategy–situation fit theory and the emotional mismatch principle, we investigated the relationship between expression and expressive suppression of academic emotions and peer acceptance in outperformance and underperformance. These research results, to some extent, validate and enrich the theory of strategy–situation fit and the principle of emotional mismatch, emphasizing the adaptive significance of individuals’ flexible use of strategies that match situational needs in their social interactions in academic environments. At the same time our results provide empirical evidence and specific guidance for adolescents to apply flexibility in using a variety of strategies for the expression of academic emotion based on situational needs to obtain better social experiences. Specifically, educators can impart to adolescents the knowledge related to the strategy of emotional regulation, cultivating an awareness in adolescents of flexibility in regulating their emotions. In addition, when adolescents outperform and underperform in comparison with others, educators can guide them to suppress their emotions if they wish to obtain more peer acceptance.

Some shortcomings in this study require attention. First, it shows that in outperformance and underperformance, adolescents usually experienced positive and negative academic emotions, respectively; however, adolescents may also experience both positive and negative academic emotions in each situation. For example, even when adolescents outperform others academically, they have regressed in comparison to their previous performance, which may lead them to experience differing valence in academic emotions. Future researchers should, therefore, continue to investigate the academic emotional experience of adolescents in outperformance and underperformance and carry out relevant research on strategies for academic emotional expression. Second, this study required participants to evaluate the acceptance level of target classmates within hypothetical scenarios—specifically in situations involving either outperformance or underperformance—rather than basing their judgments on real-life contexts. This methodological approach may limit the ecological validity of the research. Therefore, future studies should aim to enhance ecological validity in order to improve the generalizability of the findings. Third, because our participants were adolescents, children at this age are in a particularly critical stage of emotional regulation development (Theurel & Gentaz, 2018). Whether the current research results can be generalized to other groups (e.g., depression groups, other age groups, and cultural groups) remains to be seen. Researchers have found that individuals with depressive symptoms use expressive suppression more frequently (Larsen et al., 2012), and expressive suppression can effectively reduce depressive experiences (Yuan et al., 2014). Compared to other cultural groups, Asian participants typically suppress emotions more frequently (Butler et al., 2007) and produce fewer adverse outcomes (Wei et al., 2013); but emotional expression has relatively more negative impact (Soto et al., 2011). Thus, future researchers can study psychologically unhealthy populations or healthy populations with a variety of characteristics and backgrounds to explore strategies of academic emotional expression more broadly and deeply. Fourth, we examined only the costs and benefits of expression and expressive suppression of academic emotions in both outperformance and underperformance, but academic contexts are extremely rich and have many characteristics and attributes (e.g., closeness to others, peer popularity, the evaluators’ prior knowledge of the target student’s typical abilities and performance) (Graham et al., 2008; Zhou et al., 2016); therefore, exploring the impact of matching expression and expressive suppression of academic emotions with other contextual features on individuals can further validate the applicability of theories like strategy–situation fit theory and produce a more comprehensive understanding of the impact of situation on the effect of the expression and expressive suppression of academic emotions.

## 5. Conclusions

Overall, the findings of this study highlight the importance of situations involving outperformance and underperformance in the application of academic emotion regulation strategies, and validate the adaptive nature of emotional expression and expressive suppression in both types of situations; that is, compared to outperformance, expressing emotion when underperforming will result in a higher level of peer acceptance. In addition, in outperformance, the level of peer acceptance after suppressing positive academic emotions was higher than that after expressing them. In underperformance, acceptance after suppressing negative academic emotions was significantly higher than after expressing them.

## Figures and Tables

**Table 1:** Means, standard deviations and correlations for the primary variables.

		1	2	3	4	5	6	7	8
1. LSPE		1							
2. LEPE	*r*	0.08	1						
	95% CI	[−0.14, 0.32]							
3. LSNE	*r*	0.25	0.07	1					
	95% CI	[−0.01, 0.50]	[−0.20, 0.33]						
4. LENE	*r*	0.09	0.23	0.02	1				
	95% CI	[−0.18, 0.34]	[−0.04, 0.49]	[−0.25,0.27]					
5. ASPE	*r*	0.77 **	0.12	0.21	0.18	1			
	95% CI	[0.64, 0.88]	[−0.12, 0.34]	[−0.06, 0.47]	[−0.07, 0.42]				
6. AEPE	*r*	0.11	0.86 **	0.10	0.22	0.23 *	1		
	95% CI	[−0.09, 0.32]	[0.77, 0.93]	[−0.17, 0.34]	[−0.03, 0.47]	[0.01, 0.44]			
7. ASNE	*r*	0.05	−0.07	0.67 **	0.09	0.19	−0.03	1	
	95% CI	[−0.21, 0.33]	[−0.30, 0.20]	[0.48, 0.81]	[−0.18, 0.36]	[−0.06, 0.45]	[−0.27, 0.24]		
8. AENE	*r*	0.11	0.28 *	0.16	0.58 **	0.12	0.36 **	0.20	1
	95% CI	[−0.17, 0.36]	[0.03, 0.53]	[−0.15, 0.44]	[0.35, 0.78]	[−0.17, 0.41]	[0.13, 0.58]	[−0.10, 0.47]	
*M*		4.77	3.92	4.80	4.66	4.78	4.10	5.00	4.53
*SD*		1.41	1.62	1.29	1.46	1.66	1.74	1.30	1.49

Note. ** *p* < 0.01; * *p* < 0.05. LSPE = Likability after suppression of positive academic emotions; LEPE = Likability after expression of positive academic emotions; LSNE = Likability after suppression of negative academic emotions; LENE = Likability after expression of negative academic emotions; ASPE = Acceptance after suppression of positive academic emotions; AEPE = Acceptance after expression of positive academic emotions; ASNE = Acceptance after suppression of negative academic emotions; AENE = Acceptance after expression of negative academic emotions.

## Data Availability

The data supporting this study’s findings are available from the corresponding author upon reasonable request.
